# Management and radiographic outcomes of femoral head fractures

**DOI:** 10.1007/s10195-017-0445-z

**Published:** 2017-02-10

**Authors:** John A. Scolaro, Geoffrey Marecek, Reza Firoozabadi, James C. Krieg, Milton Lee “Chip” Routt

**Affiliations:** 1 University of California, Irvine, 101 The City Drive South, Building 29A, Pavilion III, 2nd Floor, Orange, CA 92868 USA; 2 University of Southern California, Los Angeles, CA USA; 3 Harborview Medical Center, University of Washington, Seattle, WA USA; 4 Rothman Institute at Jefferson Medical College, Philadelphia, PA USA; 5 The University of Texas, Health Sciences Center at Houston, Houston, TX USA

**Keywords:** Femoral head, Fracture, Hip dislocation, Pipkin, Heterotopic ossification

## Abstract

**Background:**

Femoral head fractures are uncommon injuries. Small series constitute the majority of the available literature. Surgical approach and fracture management is variable. The purpose of this study was to evaluate the incidence, method of treatment, and outcomes of consecutive femoral head fractures at a regional academic Level I trauma center.

**Materials and methods:**

A retrospective review of a prospective database was performed over a 13-year period. All AO/OTA 31C femoral head fractures were identified. A surgical approach and fixation method was recorded. Clinical and radiographic evaluation was performed for patients with 6 months or greater follow-up. Radiographs were evaluated for fixation failure, heterotopic ossification (HO), avascular necrosis (AVN) and post-traumatic arthritis.

**Results:**

We identified 164 fractures in 163 patients; 147 fractures were available for review. Treatment was operative reduction and internal fixation (ORIF) in 78 (53.1%), fragment excision in 37 (25.1%) and non-operative in 28 (19%). An anterior approach and mini-fragment screws were used in the majority of patients treated with fixation. Sixty-nine fractures had follow-up greater than 6 months. Sixty-two fractures (89.9%) proceeded to uneventful union. All Pipkin III fractures failed operative fixation. Six patients developed AVN, seven patients had a known conversion to hip arthroplasty; HO developed in 28 (40.6%) patients and rarely required excision.

**Conclusions:**

Fractures of the femoral head are rare. An anterior approach can be used for fragment excision or fixation using mini-fragment screws. Pipkin III fractures represent catastrophic injuries. Non-bridging, asymptomatic HO is common. AVN and posttraumatic degenerative disease of the hip occur but are uncommon.

**Level of evidence:**

IV—prognostic.

## Introduction

**Fig. 1:**
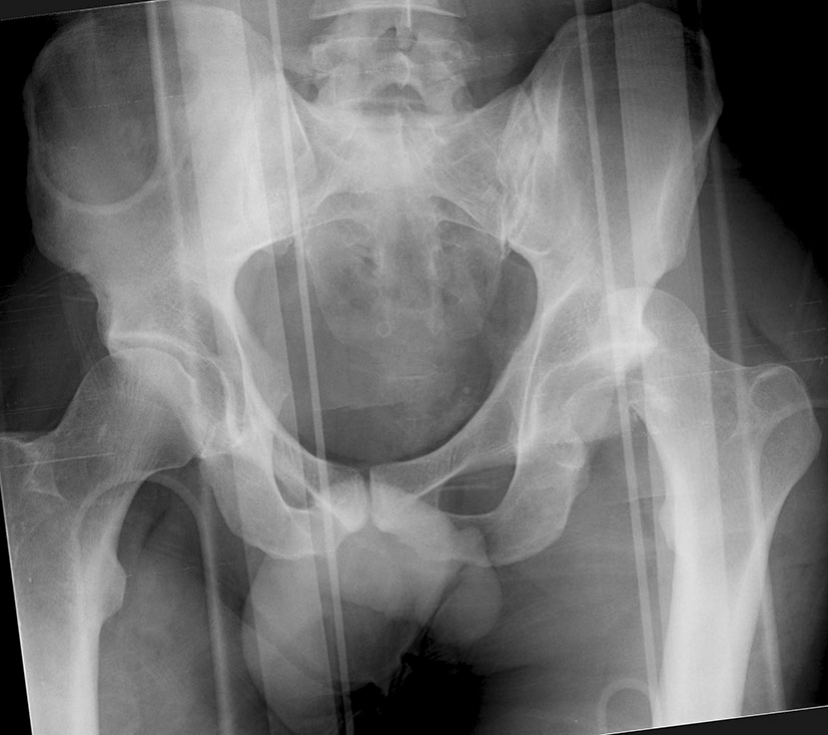
Anteroposterior (AP) pelvis radiograph of a 36-year-old male involved in a motor vehicle collision with a left femoral head fracture dislocation

Femoral head fractures are uncommon injuries. They are often the result of high-energy trauma to the hip or lower extremity, and commonly associated with a hip dislocation (Fig. 1). The infrequency of these fractures has made the study of large patient populations difficult; multiple small studies constitute the majority of the available literature on this topic. A recent systematic literature review of this heterogeneous literature was performed, and included 29 articles describing a total of 453 total fractures; definitive management data was available for only 425 fractures; 99 fractures were managed non-operatively and 326 were treated with surgery [1].

Multiple classification systems have been proposed for femoral head fractures [2–5]. No system provides absolute recommendations for fracture management or has validated prognostic value; most are purely descriptive. Femoral head fracture location, size, displacement and effect on hip stability have all been used as rationale for injury management. In fractures treated surgically, surgical approach, fragment fixation/excision and fracture fixation technique have been debated. Unfortunately, inconsistent facture classification, treatment methods and lack of comparative evidence leave few concrete conclusions. Data on the overall prognosis and long-term outcome for these injuries, specifically fixation failure, avascular necrosis (AVN), post-traumatic arthritis and conversion to total hip arthroplasty, is poorly defined.

The purpose of this study was to evaluate the initial management and documented outcomes in a large consecutive series of patients with a femoral head fracture treated at a single institution. Intermediate-term outcomes, including union, development of heterotopic ossification (HO), AVN and conversion to arthroplasty were evaluated. We hypothesized that using a standard protocol for management, surgical approach and fixation of femoral head fractures would result in low rates of fixation failure, HO and AVN. This series represents one of the largest consecutive series of patients treated for this uncommon injury.

## Materials and methods

Institutional Review Board approval was obtained for this study. A retrospective review of a prospectively collected orthopaedic trauma database at a single regional academic level-one trauma center was performed between 1 January 2000 and 1 January 2013. All patients who sustained a fracture of the femoral head (AO/OTA 31C) were identified [6]. Inclusion criteria included patients who had sustained an acute traumatic fracture of the femoral head, had available plain anteroposterior (AP) and radiograph of the affected hip, as well as a CT scan of the abdomen and pelvis before any surgical intervention had been performed. Exclusion criteria included patients with pathologic or non-acute fractures, an incomplete radiographic evaluation, or unavailable clinical documentation (initial history and physical examination, operative note, etc.).

Patient age, gender, date of injury, and date of operative procedure were collected from the medical record. Operative details included: surgical approach, surgical treatment (fragment excision or fixation) and, if applicable, hardware used. Peri-operative details such as pre-operative nerve palsy, irreducible fracture-dislocation and relevant associated injuries were also recorded. Each injury was classified according to the Pipkin system [2]. Follow up radiographs and clinical notes were collected and reviewed. Patients with ≥6 months of both radiographic and clinical follow-up were included in our final evaluation.

In this series, a uniform treatment protocol for patients with a femoral head fracture involved prompt reduction of a hip dislocation event in the emergency room. If closed reduction was unsuccessful, the patient was brought urgently to the operating room for an open reduction. Plain radiographs and computed tomography scans were obtained following reduction in all patients. In 28 cases, an intraoperative manipulative examination under anesthesia was performed when static imaging demonstrated a concentric hip joint with a non-to-minimally displaced fracture. Surgical fixation was performed in cases where hip instability or fracture displacement occurred during fluoroscopically monitored manipulation.

A total of 164 fractures of the femoral head was identified in 163 patients; 17 patients were excluded because of incomplete radiographs or clinical documentation leaving 147 fractures available for review (Table 1). Average patient age was 39.2 years (range 13–81); there were 99 males and 48 females. Fractures were classified as: Pipkin I: 40 (27%), II: 62 (42%), III: 7 (4.7%) and IV: 23 (15%); 15 (10%) fractures did not fit within the Pipkin classification system as they were primarily femoral head impaction type injuries and dissimilar to distinct femoral head fractures. Seventy-eight (53%) fractures were treated with operative reduction and internal fixation (ORIF), 37 (25%) fractures with fragment excision, 28 (19%) fractures were managed non-operatively, 3 (2%) fractures with hemiarthroplasty; one patient had an antibiotic spacer placed during initial fracture management secondary to the individual’s severe medical comorbidities and complex clinical presentation.

**Table 1:** Demographic, classification and treatment of all fractures.* ORIF* Operative reduction and internal fixation

Fractures	*n* = 147
Age	$\bar{X}$ = 39.2 years (13–81 years)
Gender	99 Males
	48 Females
Classification	
Pipkin I	40 (27%)
Pipkin II	62 (47%)
Pipkin III	7 (4.7%)
Pipkin IV	23 (15%)
Other	15 (10%)
Treatment	
ORIF	78 (53%)
Excision	37 (25%)
Non-operative	28 (19%)
Hemiarthroplasty	3 (2%)
Other	1

**Fig. 2:**
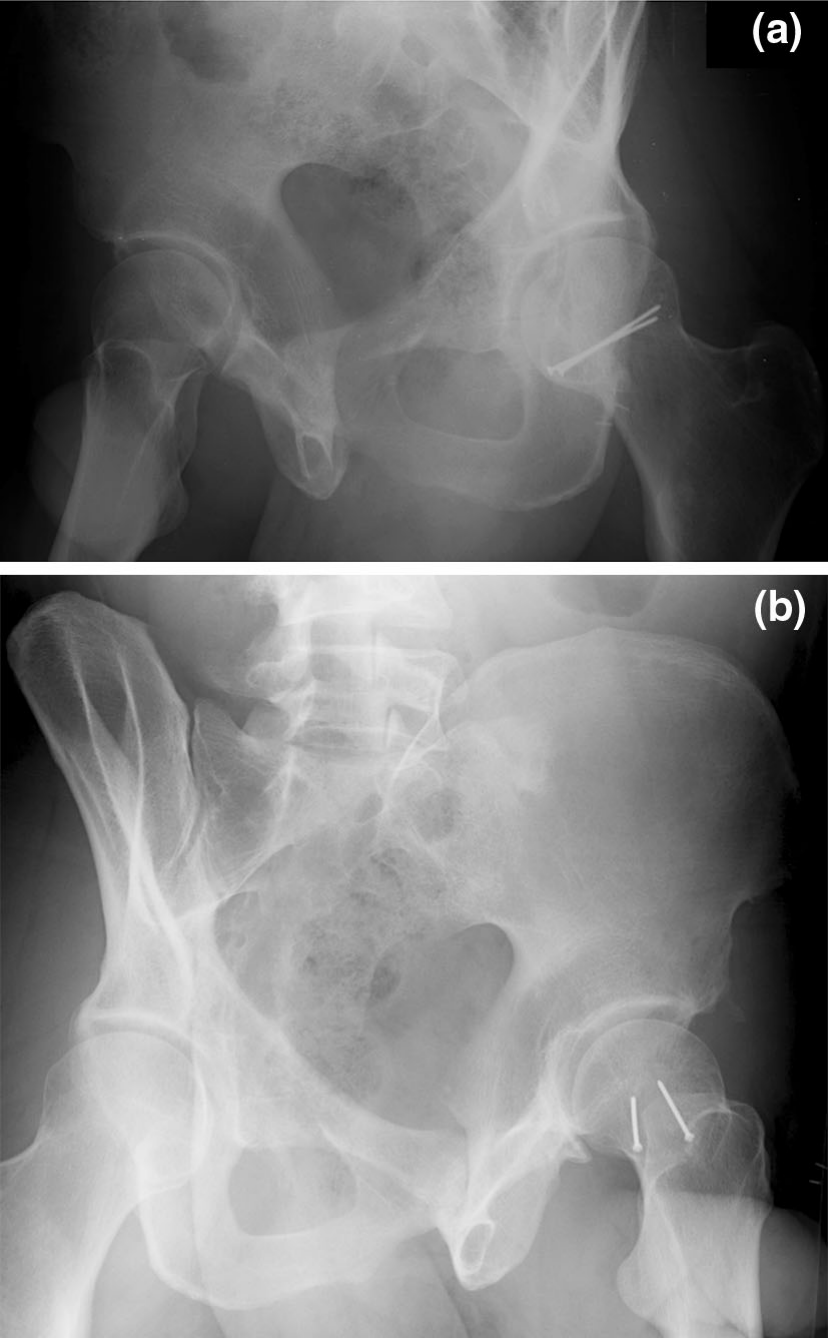
**a** Postoperative obturator and **b** iliac oblique radiographs of patient shown in Fig. 1, demonstrating fixation of the femoral head fracture with two 2.0 mm minifragment screws

Fracture fixation was performed for 78 fractures. In 76 (97%) fractures, the anterior Smith–Petersen approach was used; a tenotomy of the rectus femoris was performed as a part of this approach in 73 (96%) cases. Fixation was performed with mini-fragment (2.0 or 2.4 mm) fully threaded cortical screws, placed in lag fashion, in the majority of cases (Fig. 2a, b). Fragment and femoral neck fixation was performed initially for all Pipkin III injuries in an attempt to restore native proximal femoral anatomy. Fragment excision was performed through both anterior and posterior (Kocher–Langenbeck) approaches based on the location of the free fracture fragment. Nine patients were identified as having irreducible fracture-dislocations of the femoral head without associated posterior wall acetabular fracture [7].

Sixty-nine fractures in 68 patients had clinical and radiographic follow-up greater than 6 months (mean 12.4 months). Fracture union was defined clinically and radiographically as lack of pain with full weight bearing, fracture consolidation and need for revision or subsequent surgical procedure. The presence of radiographic sclerosis and flattening of the femoral head, as well as the presence of persistent/worsening hip pain, was used to identify AVN. Heterotopic ossification (HO) was identified on the antero-posterior pelvic radiograph and classified according to the Brooker system [8]. The location of HO was then confirmed on the lateral hip radiograph. Early failure of treatment/fixation was defined as radiographic loss of reduction, fracture displacement, femoral-acetabular joint incongruence or need for re-operation within the first 12 postoperative weeks.

## Results

Of 69 femoral head fractures, 62 (89%) with follow up equal or greater than 6 months had uncomplicated outcomes (Table 2): 42 (60%) patients in this group were managed with ORIF, 18 (26%) were treated with fragment excision, 8 patients were treated non-operatively and 1 patient was managed with primary hemiarthroplasty.

**Table 2:** Treatment, results and complications of fractures with follow-up ≥6 months

Fractures	*n* = 69
Follow up	$\bar{X}$ = 12.4 months
Treatment	
ORIF	42 (61%)
Excision	18 (26%)
Non-operative	8 (12%)
Hemiarthroplasty	1
Results	
Union	37 (88%)
Complications	
Early fixation failure	5 (12%)
Pipkin III	2
Repeat trauma	3
Avascular necrosis/post-traumatic degenerative joint disease	6 (9%)
Heterotopic ossification	28 (40%)
Brooker I	17
Brooker II	4
Brooker III	4
Brooker IV	3

One patient with follow up ≥6 months required a return trip to the operating room for superficial debriedement and irrigation of their anterior surgical wound. One patient was noted to have partial wound dehiscence after staple removal and required local wound care until uncomplicated secondary wound healing had occurred. No deep infections were noted to have occurred in either the initial patient group or in those individuals with at least 6 months of follow-up. Four patients presented with a sciatic nerve palsy following their traumatic injury; complete resolution was noted to occur in three of four patients, and was persistent in one at the time of last follow-up.

**Fig. 3:**
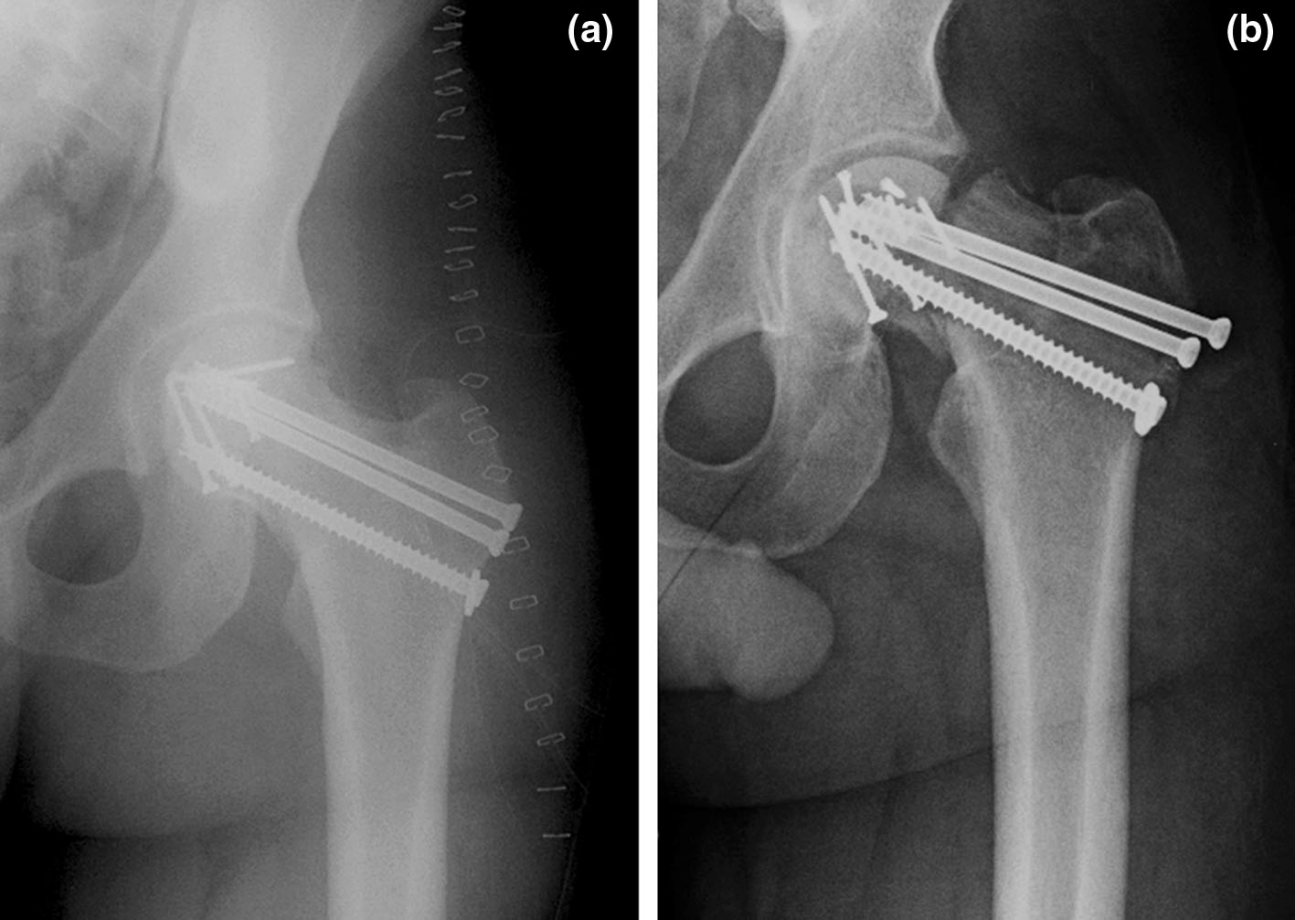
**a** Postoperative AP radiograph of a 28-year-old male who sustained a gunshot wound to the left hip and presented with a Pipkin III fracture of the femoral neck and head. Minifragment screws were used for femoral head fixation; cannulated screws were used for femoral neck fixation. **b** Three-month follow-up AP radiograph of the patient shown in **a**. Radiographs show catastrophic failure of fixation that required conversion to a total hip arthroplasty

Thirty-seven (88%) of 42 fractures treated with ORIF united without complication. In five patients, early fixation failure occurred. Three patients sustained falls within the initial post-operative period resulting in ipsilateral hip injuries, fixation failure and required conversion to hip arthroplasty. Two patients with Pipkin III fractures failed fixation within 12 weeks of initial fracture fixation (Fig. 3a, b).

**Fig. 4:**
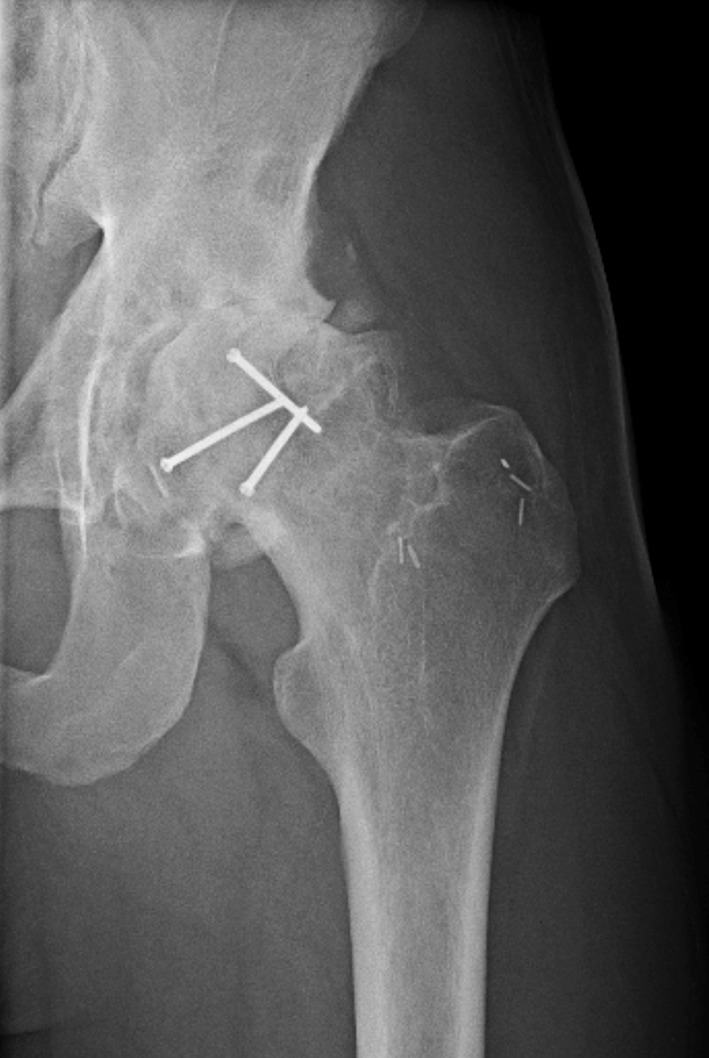
Follow-up radiograph of a 19-year-old male 4 years after left femoral head operative reduction and internal fixation (ORIF) with end stage post-traumatic arthritis in the hip joint

At last follow up (mean 12.4 months), six (8.7%) patients had developed radiographic and clinical signs of AVN or post-traumatic degenerative hip disease (Fig. 4). In total, seven (10%) patients were converted to a hip hemiarthroplasty or THA. All Pipkin III fractures (*n* = 5) with follow-up greater than 6 months proceeded to catastrophic failure or avascular necrosis (AVN).

**Fig. 5:**
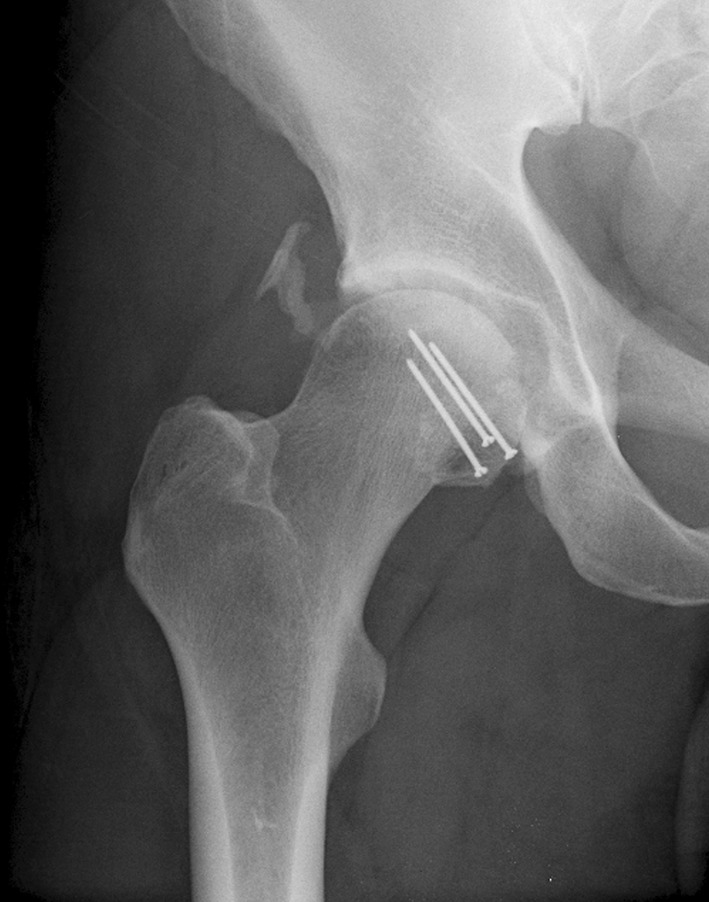
AP radiograph of a 37-year-old patient 15 months after ORIF of a femoral head fracture with non-bridging Brooker I heterotopic ossification

HO developed in 28 (40%) fractures (Fig. 5). It was classified as Brooker I in 17(60%) fractures, Brooker II in 4 (14%) fractures, Brooker III in 4 (14%) fractures and Brooker IV in 3(10%) fractures. HO was present in the anterior aspect of the hip in 23 (82%) patients, anterior and posterior in 4 (14%), and isolated to the posterior hip in 1 patient. Two (2.9%) patients required a second operation for excision of HO. Indomethacin was given in the setting of 19 fractures. Follow up data was available for 17 patients who were prescribed prophylaxis. Eight patients were found to have HO on last available radiographs. No association between the presence of HO and use of pharmacologic prophylaxis can be determined secondary to the small number of patients treated, heterogeneous group of injuries, and lack of control for medication dosage and adherence.

## Discussion

Femoral head fractures have historically been associated with poor outcomes. Pipkin stated that the nature of the injury precluded an excellent result [2]. Early outcome data reflected historic management protocols and treatment measures, primarily non-operative management or fragment excision [9, 10]. The management goals in these investigations were prompt and concentric reduction of the hip dislocation through open or closed means. More recent investigations have focused on surgical approach, fixation, and outcomes associated with different fracture types. Other common sequelae of this injury and its treatment, including HO and AVN, continue to be reported.

The infrequency of femoral head fractures makes comparative studies difficult. Swiontkowski et al. [11] compared an anterior to posterior approach for the treatment of Pipkin I and II fractures in a retrospective case matched study. To the authors’ knowledge, only two limited randomized studies on the topic exist [12, 13]. Apart from a single systematic review on the topic [1], the largest contemporary series of femoral head fractures in the literature evaluated 110 patients from multiple centers and surgeons, over a period of 36 years; only 30 patients in this group were treated with femoral head ORIF [14].

This study presents a large consecutive series of femoral head fractures treated at a single institution, primarily by the two senior authors (J.C.K. and M.L.R.). Surgical management was highly consistent throughout the study period. Some injuries were addressed by other staff members; individual fracture management was determined at the surgeon’s discretion. The majority (53.1%) of patients were treated with fragment fixation; this percentage is notably greater than other large series [14–16]. This reflects both the large number of Pipkin II fractures in our series, as well as the treatment approach we have taken to perform anatomic reduction and stable fixation to many of these injures. Radiographic and minimum 6-month follow-up data was available on 42 patients treated with osteosynthesis; this represents the largest cohort of patients treated with ORIF in the literature.

The anterior Smith–Petersen approach was utilized to address the majority of these injuries. This approach provides excellent visualization of most fractures, allows anterior femoral head dislocation, and does not disrupt the posterior based blood supply to the proximal femur. The Smith-Petersen approach has been associated with increased rates of HO [11]. Our series found that 40.6% of all patients developed radiographic signs of HO. This finding exceeds the overall 16.8% HO rate described by Giannoudis et al. in their systematic review [1], but likely represents our predominant use of the anterior approach. Despite the radiographic finding of HO, we found that it was rarely bridging and rarely limited the patient clinically. Two patients required an additional surgical procedure for excision.

Surgical fixation of femoral head fractures has been reported using multiple fixation devices [17–20]. In our series, almost all patients were treated with 2–3 2.0 mm minifragment screws placed in lag fashion across the fracture and countersunk below the chondral surface. Other implants used for internal fixation were 2.4 and 2.7 mm screws. The acetabulum functions as an internal splint for the head fragment and following anatomic reduction, we have found that use of more or larger screws is unnecessary.

Avascular necrosis has approached 25% in some series of femoral head fractures [9]. Injury to the proximal femoral blood supply is multifactorial and may occur at the time of injury or during surgical intervention. A posterior approach has been associated with greater rates of osteonecrosis than an anterior approach [1, 11, 19]. We report an overall AVN rate of 8.7% in our series of patients with minimum 6 months and mean 12 months follow-up. This rate is comparable to 11.8% reported by Giannoudis et al. [1]. Although radiographic signs of AVN were identified at 6 months in some of our patients, longer follow up may identify more patients.

Fractures of the femoral neck and femoral head represent a dual insult to the proximal femur. These injuries are the least common Pipkin type encountered across the reported literature. The severity of this fracture, and associated poor prognosis, is well recognized [21]. In our series, seven were identified over a period of 13 years. Open reduction and internal fixation was performed of both fractures in every case and fixation failure was seen in all of cases. Although other studies report treatment success, based on our experience, arthroplasty should be strongly considered for displaced fractures of the femoral neck and head.

Despite the large number of included patients, less than 50% had recorded follow-up greater than 6 months. Our center provides care to patients within a large geographical region who follow up with other providers or are unable to travel back to our clinics. Follow up information was also acquired from review of the medical records and archived digital image system. Future work will focus on obtaining longer-term clinical and radiographic follow-up data on their injuries. Functional outcome data, including collection of validated functional outcome scores would add to our results. Improved subgroup and comparative analyses would also be helped with this information.

In conclusion, femoral head fractures are infrequently encountered injuries. Urgent closed or open concentric reduction of the hip joint should occur. Surgical treatment can successfully be performed using an anterior hip approach in most cases with minifragment lag screw fixation. Pipkin III fractures are a unique subset of injuries that may not be amenable to successful surgical fixation secondary to high rates of fixation failure or AVN. Non-bridging HO is common following operative intervention through an anterior approach but rarely requires a second surgical procedure for removal. Fracture union and progression to uneventful radiographic healing should be expected.

## References

[1] Giannoudis PV, Kontakis G, Christoforakis Z, Akula M, Tosounidis T, Koutras C (2009). Management, complications and clinical results of femoral head fractures. Injury.

[2] Pipkin G (1957). Treatment of grade IV fracture-dislocation of the hip. J Bone Joint Surg Am.

[3] Brumback RJ, Kenzora JE, Levitt LE, Burgess AR, Poka A (1987) Fractures of the femoral head. Hip 1987:181–206 3546215

[4] Yoon TR, Rowe SM, Chung JY, Song EK, Jung ST, Anwar IB (2001). Clinical and radiographic outcome of femoral head fractures: 30 patients followed for 3–10 years. Acta Orthop Scand.

[5] Tonetti J, Ruatti S, Lafontan V, Loubignac F, Chiron P, Sari-Ali H, Bonnevialle P (2010). Is femoral head fracture-dislocation management improvable: a retrospective study in 110 cases. Orthop Traumatol Surg Res.

[6] Marsh JL, Slongo TF, Agel J (2007). Fracture and Dislocation Classification Compendium-2007: orthopaedic Trauma Association classification, database and outcomes committee. J Orthop Trauma.

[7] Mehta S, Routt ML (2008). Irreducible fracture-dislocations of the femoral head without posterior wall acetabular fractures. J Orthop Trauma.

[8] Brooker AF, Bowerman JW, Robinson RA, Riley LH (1973). Ectopic ossification following total hip replacement. Incidence and a method of classification. J Bone Joint Surg Am.

[9] Epstein HC (1974). Posterior fracture-dislocations of the hip; long-term follow-up. J Bone Joint Surg Am.

[10] Epstein HC, Wiss DA, Cozen L (1985). Posterior fracture dislocation of the hip with fractures of the femoral head. Clin Orthop Relat Res.

[11] Swiontkowski MF, Thorpe M, Seiler JG, Hansen ST (1992). Operative management of displaced femoral head fractures: case-matched comparison of anterior versus posterior approaches for Pipkin I and Pipkin II fractures. J Orthop Trauma.

[12] Chen ZW, Zhai WL, Ding ZQ, Lian KJ, Kang LQ, Guo LX, Liu H, Lin B (2011). Operative versus nonoperative management of Pipkin type-II fractures associated with posterior hip dislocation. Orthopedics.

[13] Lin D, Lian K, Chen Z, Wang L, Hao J, Zhang H (2013). Emergent surgical reduction and fixation for Pipkin type I femoral fractures. Orthopedics.

[14] Tonetti J, Ruatti S, Lafontan V, Loubignac F, Chiron P, Sari-Ali H, Bonnevialle P (2010). Is femoral head fracture-dislocation management improvable: a retrospective study in 110 cases. Orthop Traumatol Surg Res.

[15] Marchetti ME, Steinberg GG, Coumas JM (1996). Intermediate-term experience of Pipkin fracture-dislocations of the hip. J Orthop Trauma.

[16] Lederer S, Tauber M, Karpik S, Bogner R, Auffarth A, Resch H (2007). Fractures of the femoral head. A multicenter study. Unfallchirurg.

[17] Butler JE (1981). Pipkin Type-II fractures of the femoral head. J Bone Joint Surg Am.

[18] Hermus JP, Laan CA, Hogervorst M, Rhemrev SJ (2005). Fixation of a Pipkin fracture with bio-absorbable screws. Case report and a review of the literature. Injury.

[19] Stannard JP, Harris HW, Volgas DA, Alonso JE (2000). Functional outcome of patients with femoral head fractures associated with hip dislocations. Clin Orthop Relat Res.

[20] Homma Y, Miyahara S, Mogami A, Morohashi I, Baba T, Kaneko K (2014). Percutaneous screw fixation for a femoral head fracture: a case report. Arch Orthop Trauma Surg.

[21] Hougaard K, Thomsen PB (1988). Traumatic posterior fracture-dislocation of the hip with fracture of the femoral head or neck, or both. J Bone Joint Surg Am.

